# Letter to Editor: Implementation of Patient and Family Communication Model in Different Settings

**DOI:** 10.4103/JQSH.JQSH_3_20

**Published:** 2020-02-14

**Authors:** Abdul Rahman Jazieh

**Affiliations:** Department of Oncology, Ministry of National Guards Health Affairs, Riyadh, Saudi Arabia

Dear Editor,

We would like to thank our colleague, Dr Kamal, for her interest in our publication about the communication model with patients and families and for her feedback about the topic.[1]

Although our manuscript gives an example of the model use in a setting with large families, this model can be applied in any setting with any size families because there are core values maintained in the model. The main value is keeping patients in the center of communication and decision-making, thus maintaining their rights in selecting the most responsible family member and the ability to change their decision whenever they want. If the patient cannot make decision, such as being a minor or having mental status changes, then the model defaults into the prevalent regulations and laws in appointing next of kin.[2]

The model does not conflict with other approaches that are implemented in some countries such as living will and advanced directives as the patients can decide about their wishes in an official way, but the model gives a practical alternative in settings where these concepts are not applied.

We were concerned that patients and family members may not accept the concept, but through multiple plan-do-study-act (PDSA) cycles and hundreds of encounters, we found general acceptance in our populations.[3]

Although the model is easily adaptable to different settings, this does not mean that there will be no challenges related to local cultures, societal norms, and maybe laws and regulations. Therefore, having a real-world experience of adapting the model to different settings and sharing the lessons learned about the challenges encountered and the pros and cons of the model in that setting will be of value to others.

We look forward to hearing about real-world applications of the model and learning how we can make the communications with our patients and their families better to enable us to provide better patient-centered care.
